# Peer involvement and cross-sector efforts in establishing integrated treatment of hepatitis C virus infection for people with substance use disorders: experiences from Norway

**DOI:** 10.1186/s13011-019-0245-y

**Published:** 2019-12-21

**Authors:** Ole Jørgen Lygren, Ronny Bjørnestad, Else-Marie Løberg, Martine Lepsøy Bonnier, Vibeke Bråthen Buljovcic, Kjell Arne Johansson, Lars T. Fadnes, for the INTRO-HCV Study Group, Christer Frode Aas, Vibeke Bråthen Buljovcic, Fatemeh Chalabianloo, Jan Tore Daltveit, Silvia Eiken Alpers, Lars T. Fadnes, Trude Fondenes Eriksen, Per Gundersen, Velinda Hille, Kristin Holmelid Håberg, Kjell Arne Johansson, Rafael Alexander Leiva, Siv-Elin Leirvåg Carlsen, Martine Lepsøy Bonnier, Lennart Lorås, Else-Marie Løberg, Mette Hegland Nordbotn, Maria Olsvold, Christian Ohldieck, Lillian Sivertsen, Hugo Torjussen, Jørn-Henrik Vold, Jan-Magnus Økland, Tone Lise Eielsen, Nancy Laura Ortega Maldonado, Ewa Joanna Wilk, Ronny Bjørnestad, Ole Jørgen Lygren, Marianne Cook Pierron, Olav Dalgard, Håvard Midgard, Svetlana Skurtveit, Peter Vickerman

**Affiliations:** 1 ProLAR Nett, Søgne, Norway; 2 Bergen Addiction Research Group, Department of Addiction Medicine, Haukeland University Hospital, Avdeling for rusmedisin, Postboks 1400, 5021 Bergen, Norway; 3 Department of Clinical Psychology, Medicine, University of Bergen, Bergen, Norway; 4 Department of Medicine, Division of Psychiatry, Haukeland University Hospital, Bergen, Norway; 5 NORMENT Center of Excellence, Haukeland University Hospital, Bergen, Norway; 6 Agency for mental health and substance abuse services, Bergen municipality, Norway; 7 Department of Global Public Health and Primary Care, University of Bergen, Bergen, Norway

**Keywords:** Chronic hepatitis C, Opiate substitution treatment, Integrated health care, Substance abuse treatment centers

## Abstract

**Background:**

For people with opioid dependence in Norway, chronic hepatitis C virus (HCV) infections contribute to high mortality and high morbidity. Around 50% of patients in medically assisted rehabilitation (MAR) have been shown to have HCV, and the current prevention and control efforts have been mostly unsuccessful. Thus, there is a need for new strategies for people-centred service delivery and innovative methods to improve health outcomes.

**Methods:**

Over the last few years, the city of Bergen, Norway, has developed a cross-sector collaboration with substantial peer involvement in research and health provision related to substance use. User group representatives for people receiving MAR, addiction medicine health personnel, infectious disease specialists, policy makers in the municipality, low-threshold health care centres for people with substance use disorders in Bergen Municipality and researchers in the INTRO-HCV project have made concerted efforts in this regard. We will present here some of the strategies and steps we have taken.

**Results:**

We have established an integrated HCV treatment scheme for people who inject drugs or who have opioid dependence. More than 800 persons have been tested for HCV within these frames, and more than 250 persons have been given treatment for HCV within the project. The integrated treatment of HCV is offered both in MAR outpatient clinics, municipal low-threshold healthcare centres, and local and regional prisons. The preliminary results indicate an increase in HCV treatment uptake among those receiving integrated treatment (96% initiating treatment compared to 75%). The user group organisation ProLAR Nett has established an outreach service to screen for HCV, increase awareness and reduce the proportion of people unknowingly living with HCV while informing and motivating people to receive treatment. Together with the other stake holders, peer user group, health care, research planning, concert events, and policy panels have been held.

**Conclusions:**

Peer involvement seems to have increased testing rates for HCV and acknowledgment of its importance. This seems to have improved health care for people with opioid dependence in Bergen over the last few years, particularly relating to the treatment of HCV. These experiences might be helpful in the planning of integrated policies in other settings that seek to eliminate the HCV endemic.

## Background

In addition to overdose deaths [1], people with opioid dependence in Norway have suffered from high co-morbidities including chronic hepatitis C virus infections (HCV) [2]. HCV and hepatitis B are among the leading causes of liver disease and transplantation worldwide [3–5], and HCV substantially increases the risk of severe complications such as liver failure and early death [6, 7]. Among people with HCV, about a third develops severe hepatic complications within three decades [8]. Among people with HCV and opioid dependence, liver disease and overdose have the same case fatality risk after the age of 50 years [9].

Around 50% of the patients receiving medically assisted rehabilitation (MAR) are infected by HCV according to reports from outpatient clinics that provide opioid agonist therapy (OAT) as part of the MAR programme in Norway [10]. The mean age of MAR patients in Norway is 42 years, and the population is aging [11]. Many patients have had HCV for more than 20 years. Reaching these patients with treatment is of critical importance to avoid early deaths and reduced quality of life, as well as for preventing large costs for future treatment of end-stage liver disease. Over the last several years, the development of highly effective tablet-based direct-acting antiviral medications, has radically changed HCV treatment [12, 13]. These antivirals are usually curative within 8 to 12 weeks and most experience few side-effects. However, utilisation of standard care is low among people in need of HCV treatment in Norway and the majority have until recently remained untreated [14]. The high burden of HCV is among the greatest challenges among people who inject drugs. This group is also underrepresented in clinical research.

Despite recent advances, the prevention and control of HCV remains difficult. The World Hepatitis Summit in São Paulo expressed concern over the lack of integrated approaches, acknowledging the need for innovative strategies for people-centred service delivery [15]. An editorial from 2017 points out that there is very little research on user involvement in substance use related research [16]. Still, there are several good arguments for why user involvement could be important. People who might be affected by the research should be listened to and involved when possible, and user representatives experiences might improve how study participation is perceived. This is important for research quality and for reducing loss to follow-up. User involvement might also aid in study implementation and in putting the research into context and increasing its relevance.

Since 2014, Bergen has gradually increased peer involvement and established an integrated treatment scheme for HCV in order to improve the health of people with opioid dependence and people who inject drugs in Bergen, Norway. We will here present some of the strategies and steps we have taken and some of the results of this work.

## Methods

**Fig. 1:**
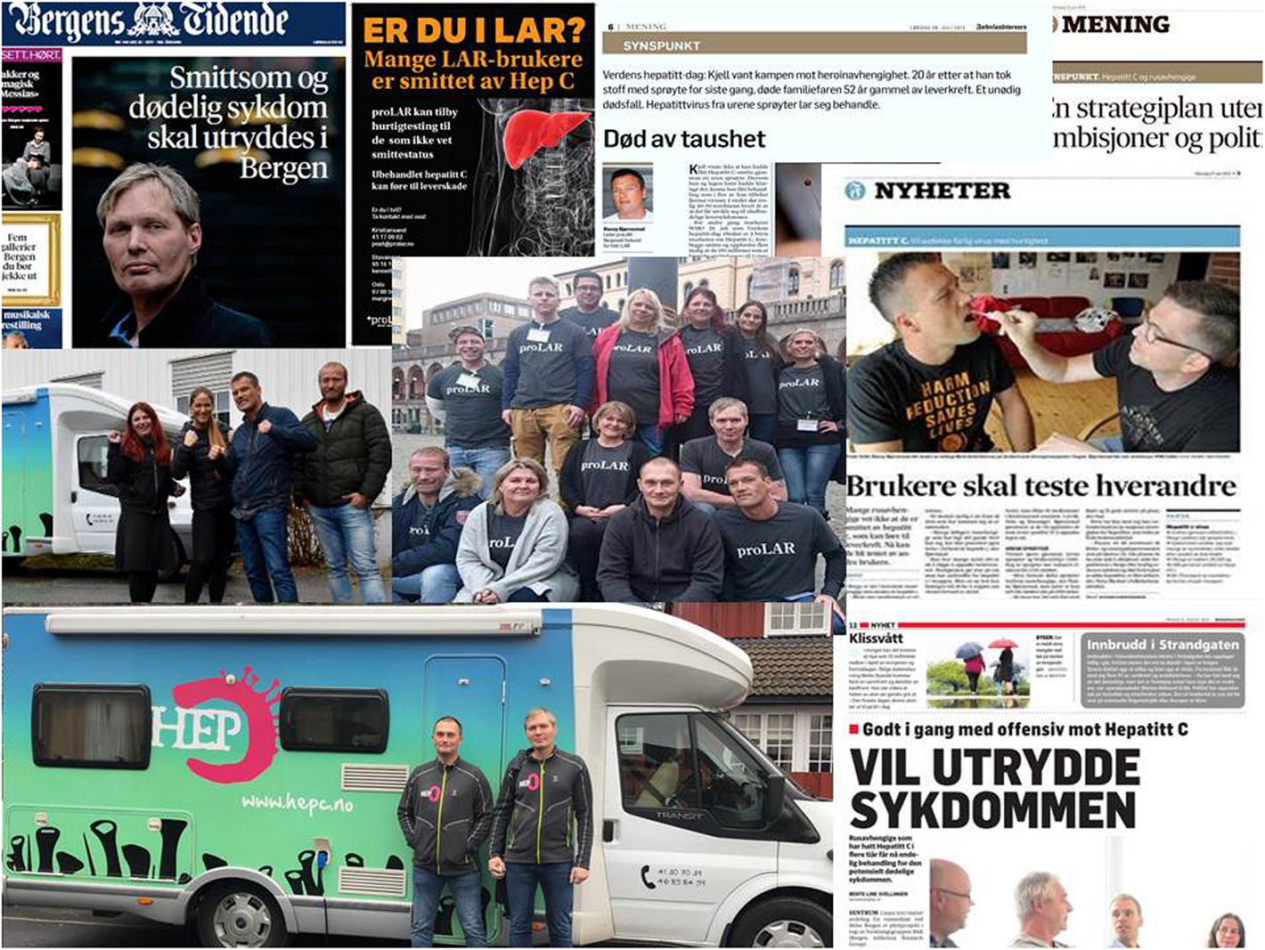
Photos from some of the responses mentioned in the paper including some of the newspaper stories and the outreach bus used («*Hepatittbussen*»)

We have established a cross-sector collaboration between peer groups, primary and secondary health care, policy makers and researchers with peer involvement as a core component. We will here summarise the concerted efforts and the role of the different stakeholders (see also Table 1 and Fig. 1).

**Table 1:** Table summarising the collaborative and concerted efforts

	Information interventions	Outreach interventions	Integrated health care
Interventions towards users	User group seminars led by user group representatives together with MAR clinics and the INTRO-HCV project Information brochure developed by user group representatives and the INTRO-HCV project reaching 6000 persons	Establishing of outreach bus («*Hepatittbussen*») led by user group representatives in ProLAR Nett to diagnose people with hepatitis C, informing and motivating for treatment	Establishing an integrated health care response in collaboration between user group representatives, MAR clinics, internal medicine specialists, municipal health care clinics and researchers coordinated by INTRO-HCV project. The integrated assessment and treatment has reached 800 persons in MAR outpatient clinics, municipal health care clinics, and local and regional prisons.
Interventions towards health care workers	User group members working in MAR clinics and health care seminars led by MAR clinics, the INTRO-HCV project and user group representatives		Research planning seminars led by INTRO-HCV project in collaboration with user group representatives, MAR clinics, and municipal health care officials. Around 10 seminars have been held.
Policy oriented interventions and public communication	The concert «*Lever4Livet*» reached a general population group in the Bergen area	Policy panels led by municipal health care policy makers with user group representatives	Several mass media communications and have reached a large population group in the Bergen area

### Integrated specialised health care and low thresholds

The Department of Addiction Medicine at Haukeland University Hospital in Bergen, Norway, developed the Bergen model of MAR, which is an integrated treatment and care model for patients with opioid dependence. Firstly, the evidence indicates that OAT is the preferred treatment with the best outcomes for people with opioid dependence [17]. Secondly, lowered barriers to OAT access are expected to limit the illegal opioid market and thus reduce the recruitment of new opioid users, and reduce the number of opioid overdose deaths [18]. The model is based on several principles and treatment elements, and the services are decentralised and community-based in order to optimise access for patients often hard to reach. In Bergen, several MAR outpatient clinics have been established across the city. Every month, the MAR clinics have a mean of 12 outpatient clinic visits per patient and serve approximately 500–600 patients. Patients are followed up by a multidisciplinary team, and each of the MAR outpatient clinics is staffed by teams specialised in addiction healthcare, including physicians, nurses, social workers and clinical psychologists. These teams provide both non-pharmacological and psychosocial follow-up closely linked to pharmacological interventions. In addition, user group representatives are also being employed to aid in follow-up at the clinics. Health care and social workers meet many of the patients on an almost daily basis with observed intake of the MAR medications [19]. This group of patients often has multiple severe drug disorders and significant co-morbidity and has only to a limited degree been able to access other standard health care services. Treatment is given with frequent clinical follow-up often involving outpatient visits several days per week. Further, consultations and follow-up have been organised using a drop-in approach and limited use of scheduled appointments and urine screening tests. Together with municipal low-threshold health care centres, a programme has deployed nasal naloxone for treatment of overdoses. A collaboration has also been established with infectious disease specialists at the Department of Internal Medicine at Haukeland University Hospital to facilitate the integrated treatment of HCV (see more details below). As foundation for the integrated treatment, competence building among clinicians and user groups and flexibility to adapt to initially different routines have been emphasised. The Department of Addiction Medicine has further established two advisory boards in order to include user experiences in the development of the services. The advisory boards meet every seventh week with directors and administrative leaders. One of the outcomes from this has been the employment of people with user experiences in the MAR clinics in order to improve the user orientation of the services.

### Integration of health care and research

As part of this treatment model in Bergen, we are now testing further scale-up strategies for the integrated treatment of people with HCV with opioid dependence or people who inject drugs in order to end the HCV endemic among people who inject drug in Norway. More specifically, the aim is to improve the health of a vulnerable group while reducing the risk of disease transmission within the population. Further, we aim to gather evidence on the health of people who inject drugs which traditionally have been difficult to obtain, including the incidence of HCV and how treatment approaches impact HCV incidence [20]. The Bergen Addiction Research group (BAR) has initiated the INTRO-HCV study comparing the efficacy of integrated treatment of HCV within the MAR clinics and the low-threshold health care centres [21, 22]. INTRO-HCV is a multicentre, randomised controlled clinical trial without pharmaceutical industry involvement. The intervention involves integrating diagnostic and treatment follow-up for HCV treatment into the MAR treatment that patients receive in outpatient clinics including testing for HCV, counselling and treatment evaluation and treatment delivery. For people followed up in municipal low-threshold health care centres, integrated treatment will be given in a more “tailored” and individualised manner, linked to the health care they receive from the municipal centres. The main endpoint is sustained virologic response of HCV at 12 weeks after treatment and participants will be screened for hepatitis C at least annually for the total study duration between 2017 and 2021 (a detailed trial description is found in the study protocol [22]). The surveillance also includes several health indicators such as quality of life, mental health symptoms, and the presence of other diseases in need of treatment. To triangulate the outcome data, we will also analyse Norwegian registry data (including the Norwegian prescription registry) to assess uptake of treatment for hepatitis C infection. This study will provide evidence for the relative advantages and disadvantages of integrating a treatment programme for HCV into MAR or low-threshold care compared to standard care aiming to increase access to treatment and to improve treatment adherence. If the integrated treatment structure is found to be safe and efficacious, it can be considered for further scale-up.

### Peer involvement

The ProLAR Nett user group members have had a strong focus on HCV. With this focus, they have had an important role as research partners and worked closely with clinicians and policy makers. The user group organisation has taken the initiative and establish and organise an outreach bus service (“*Hepatittbussen*”) to test for HCV, focusing on awareness with an objective of reducing the proportion of people unknowingly living with HCV while informing and motivating for treatment. The bus has been used since 2018 and is planning to reach 1500 people with information and screening for HCV [23]. The background for using a mobile treatment clinic is to be a low-threshold service in order to improve access for people struggling to make use of regular hospital clinic services. The service has gotten attention in the mass media and has contributed to increasing awareness around HCV. The bus is staffed with a trained nurse and user group personnel, and it uses instant antibody screening tests. The user involvement is also regarded as important to improve how the services reach the user group. To increase knowledge on HCV, the user group organisation has built a web page with extensive information and short films presenting stories of people living with HCV (*hepc.no*). The web page also includes a contact form for people to send questions to a gastroenterologist/hepatologist. An information brochure that is given to people with substance use has been developed by ProLAR Nett together with INTRO-HCV project (see Additional file 1). Further, the user organisation has also contributed to several workshops with the European Liver Patient Association [24].

### Collaboration across sectors and delivery platforms

A close cooperation has also been established between the Department of Addiction Medicine and the Agency for Mental Health and Substance Abuse Services in Bergen municipality, which is responsible for the care in low-threshold health care centres in Bergen providing care for people with substance use disorders. In 2017, the policy makers in Bergen adopted an action plan for preventing overdose deaths. One of the important measures in this plan is to offer testing and integrated treatment of HCV to people who inject drugs. According to Norwegian guidelines, OAT is given by secondary health care while people with opioid dependence at high risk of overdose are given health care mostly by primary health care providers, including municipal providers. In many Norwegian settings, there is a risk of uncoordinated health care in these cases. In Bergen, municipal providers and secondary health care have worked together with peer involvement to avoid such gaps. As a political response to these challenges, the Bergen community council established a drug policy advisory board where they elected a group consisting of representatives from civil society including members of user organisations, next-of-kin organisations, and non-governmental organisations working with substance use disorders. The advisory board meets every six weeks with local politicians. The advisory board is invited to give guidance to on-going drug-related debates based on their experiences.

## Results

To date, the outreach bus has reached around 500 persons and 200 of these persons have been tested for HCV with rapid tests for HCV RNA in addition to being assessed for liver fibrosis with mobile elastography. Many of these have been linked up to further follow-up for prompt HCV treatment. The information brochure has been provided to eight MAR clinics and low-threshold clinics in Bergen and directly to people living with HCV and has reached 2000 users in Bergen and 4000 elsewhere in Norway. Through the web page, contact persons have been contacted by substance users, family members and healthcare professionals wanting to learn more about treatment options and prognosis, and this contributed to debunking many myths around HCV. The user union representatives have been interviewed more than ten times in national and local media such as newspapers and television and have thus contributed to awareness about the barriers to access to treatment.

ProLAR Nett started with user to user information courses and conducted around ten peer seminars at the MAR clinics and contributed to health care seminars led by the clinics and the research project INTRO-HCV. User group representatives also gave a number of presentations to different professional groups including policy makers and healthcare and social workers. The user group and INTRO-HCV also arranged a concert to communicate to the people in Bergen with famous artists including Phil Spalding (“*Lever4Livet*” [*Living/Liver4Life*]).

The involvement of peer representatives in the advisory board of the Bergen Municipality Council contributed to the restructuring of the former injection room to allow for safer substance use patterns such as smoking, nasal and oral intake rather than only injecting drugs. Further, the injection room initially offered observation of only heroin use, but after the change offered observation also to people using other drugs or a combination of substances. Bergen Municipality and policy makers have also developed an extensive policy document on the management of substance use [25].

Through the INTRO-HCV project, around 800 patients receiving OAT have been tested for HCV and other morbidities. More than 250 persons found to have chronic HCV have been treated within the project. The preliminary results indicate an increase in HCV treatment uptake among those receiving integrated treatment (96% initiating treatment compared to 75%). The assessment has also contributed to diagnosing infections such as syphilis, low thyroid levels, vitamin deficiencies, etcetera.

During the implementation of the efforts mentioned we faced several challenges that we managed to overcome (see Table 2).

**Table 2:** Lessons learned during implementation of efforts focusing on testing and treatment of hepatitis C virus (HCV)

	Periods with difficulties in accessing HCV treatment	Initial difficulties in reaching people who inject drugs	Rotation in clinical staff
Description of situation	Until February 2018, Norwegian guidelines for HCV made treatment available for only approximately half of the people with chronic HCV. The other half were required to wait during monitoring until liver fibrosis had developed. Both user groups, clinicians and researchers working with HCV worked hard to change this policy	Initially, several patients were less open for testing of HCV. This might partly have been related to some initial tension in the patient-clinician relationship, as many patients were not satisfied with the choice of opioid agonist therapy they received and the follow-up requirements for the opioid	Some of the clinical staff groups such as physicians rotated frequently between different clinical sections. This made it more difficult to plan clinical patient contact and meetings with these groups
Impact of challenges	The guidelines caused substantial frustration for people living with HCV as they were impatient to initiate treatment. It threatened a good patient-clinician relationship as clinicians were not able to provide HCV treatment to people who wanted this	The situation contributed to an initial slower start in testing of HCV, particularly during the first few months	This situation also made implementation of new routines more challenging as there was often a need for frequent training and provision of information
Efforts to overcome the challenges	The user group involvement helped patients living with HCV understand that the delay in making treatment available was due to policy that the clinicians were required to adhere to. This reduced tension	Efforts from highly motivated and patient-centred nurses together with user group involvement contributed to an improved patient-clinician relationship making testing and treatment of HCV more feasible and efficient	Through a combination of shifting of some tasks from clinicians with frequent rotation to clinicians with higher degree of stability in addition to frequent provision of information and training, we managed to achieve good continuity and quality of care

## Discussion

To optimise health services for people with substance use disorders, new approaches seem to be required, and using a model of health care focusing on interdisciplinarity and accessibility with decentralised and frequent follow-ups have been suggested to be effective [26–28]. Our collaboration between healthcare workers, researchers and user group members has been experienced as fundamental in order to increase trust and mutual understanding, to increase knowledge among healthcare workers, policy makers and users, and to break down barriers and stigma. Some of the key components of our efforts are the integrated health care response, establishing of user group panels, holding health care and user seminars, arranging concerts, use of mass media, and use of an outreach bus service. These efforts have substantially increased testing and treatment for HCV, but also improved identification of other health problems in need of follow-up and treatment. User involvement in roles as research collaborators have contributed to aiding feasibility of study implementation and also helps us in putting the research into context.

## Conclusion

In summary, the Bergen collaborative model has used new approaches aiming to reduce opioid dependence-related morbidity such as HCV. This model has contributed to improving health care for people with opioid dependence in Bergen over the last few years, and these experiences might be helpful in other settings on the path to eliminate the HCV endemic. User involvement has been an essential component in this regard. Although it is too early to draw a conclusion regarding the full impact of the Bergen MAR model with substantial peer involvement, the preliminary data are promising. Perhaps the Bergen model and user group involvement might inspire the development of adapted steps leading to improved health outcomes among people with substance use disorders – not only for fighting HCV, but also for reducing opioid dependence-related morbidity and deaths.

## Supplementary information

**Additional file 1.** **Brochure**: Brochure providing information on hepatitis C virus made by the user group ProLAR Nett together with INTRO-HCV research group.

## Data Availability

See supplementary data.

## Abbreviations

HCV: Hepatitis C infection, medically assisted rehabilitation (MAR)
OAT: opioid agonist therapy, MAR
