# Supplementary material for: Peer involvement and cross-sector efforts in establishing integrated treatment of hepatitis C virus infection for people with substance use disorders: experiences from Norway
Source: Subst Abuse Treat Prev Policy. 2019 Dec 21;14:58. doi: 10.1186/s13011-019-0245-y (PMC6925489; doi:10.1186/s13011-019-0245-y)
Supplement: Supplementary file 1 — Additional file 1. Brochure: Brochure providing information on hepatitis C virus made by the user group ProLAR Nett together with INTRO-HCV research group. [file 13011_2019_245_MOESM1_ESM.pdf]

Å få behandling

Fra 2017 har norske sykehus mulighet til å behandle de aller fleste som har hepatitt:
Alle med genotype 1 og 4 kan få ny behandling uten interferon, men pga økonomi er det fortsatt behandlingskriterier basert på grad av leverskade for de med genotype 2 og 3. Her gjelder de gamle reglene om at sykdommen må ha gitt en viss leverskade før behandling med nye medikamenter gis.
I Norge har ca 40% av alle smittede genotype 1.

Testing og behandling er gratis.

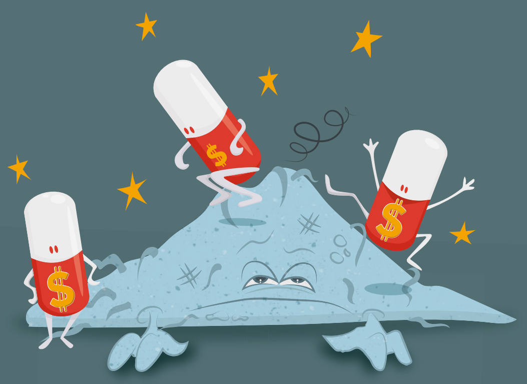

Behandlingen som gis per i dag har mindre bivirkninger enn før, og gis vanligvis som 8 – 16 ukers tablettkur.
Resultatene er veldig gode og de fleste blir virusfri etter endt behandling.
Ta kontakt med din fastlege eller LAR-poliklinikk!

Design av TONJE GJELSTEN / PXLS.no   Prosjektet "Det er din lever" er laget i samarbeid med INTRO HCV

Spør oss om råd

proLAR som brukerorganisasjon på rusfeltet har i flere år arbeidet for at flere rusavhengige skal få behandling for sin hepatitt.
Vi har blant annet utgitt info materiell og hatt kurs om hepatitt C til brukere flere steder i landet.
Dersom du ønsker å ta en prat med oss før du kontakter helsepersonell, stiller vi gjerne opp for deg med gode råd angående hepatitt C og LAR behandling.
Vi tilbyr også hurtigtest av hepatitt C antistoff.
I 2017 vil vi også utarbeide egen web side med relevant informasjon om hepatitt C viruset.
Se www.hepc.no

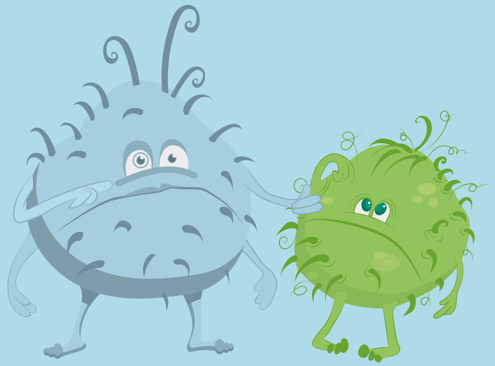

Ta kontakt med

Ole Jørgen Lygren i proLAR
Tlf: 468 58 459/ mail: ole@prolar.no
Besøksadresse:
Østre Muralmenningen 7, AFR helse Bergen

proLAR
NASJONALT FORBUND FOR FOLK I LAR

Det er *DIN* LEVER

Informasjon om Hepatitt c

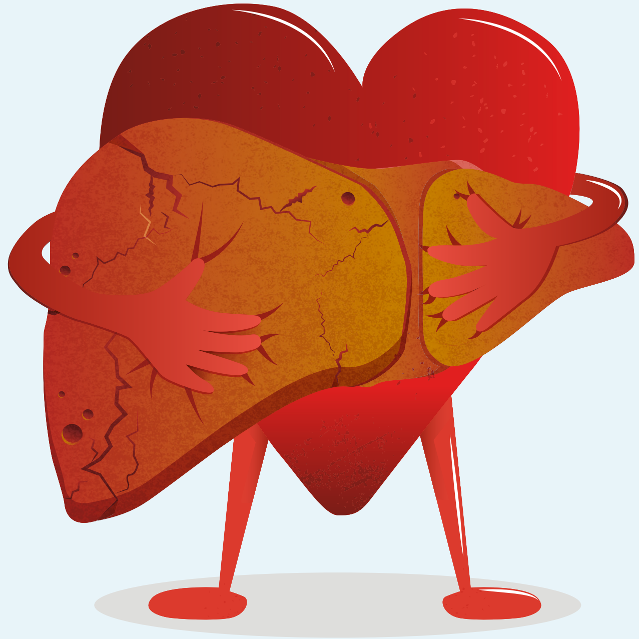

proLAR
NASJONALT FORBUND FOR FOLK I LAR

brosjyreHepCBergen.indd 1   07.04.2017 11:57:59

Halvparten av de som har tatt stoff med sprøyte har kronisk hepatitt C. Viruset kan både forebygges og behandles. Fastlegen din og LAR-klinikken kan gi deg mer informasjon.

Hva er hepatitt C

Siden hepatitt C er regnet som en allmennfarlig smittsom sykdom av Folkehelseinstituttet, skal du i utgangspunktet ikke betale egenandel for testing og behandling av dette. Du kan gjerne minne fastlegen på dette.
Viruset angriper leveren og kan utvikle seg til kronisk infeksjon, i verste fall leversvikt og leverkreft.
I Norge har så mange som 14 000 mennesker kronisk hepatitt C. Smittevei i Norge er for de fleste bruk av urene sprøyter, filtre og kokekar.
Blod må komme i kontakt med blod for at viruset skal smitte. Rusavhengige som injiserer er derfor en særlig utsatt gruppe.

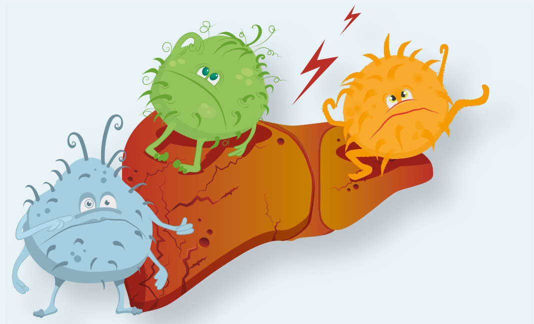

Det finnes flere ulike typer hepatitt C -virus. De kalles genotyper og mest vanlig i Norge er genotype 1, 2 og 3.
Verdens helseorganisasjon WHO anslår nå at så mange som 85 millioner mennesker er infisert globalt.

Ta en test

Man kan være smittet uten å vite om det.
Det er derfor veldig viktig at du tar kontakt med din fastlege eller ved poliklinikken.
Be om å ta en test for hepatitt C (HCV-RNA) som kan gi deg svar på om du er smittet og hvilken genotype du har.
Dersom du er smittet og har kronisk hepatitt C, må du be om henvisning til sykehuset som kan utføre leverundersøkelse med ultralyd.

proLAR tilbyr hurtigtest av hepatitt antistoff.

Symptomer på akutt hepatitt

- Tretthet
- Mørk urin
- Vekttap
- Lite matlyst
- Smerte i mage
- Kløe
- Hodepine

Hepatitt C kan gi ulike symptomer og det oppfordres til å ta kontakt med lege for å avklare om noe kan gjøres.

Risiko

Ubehandlet hepatitt C kan føre til store leverskader. En av tre som har kronisk hepatitt vil utvikle alvorlig leversykdom *(skrumplever)*, men ofte først i 50 -60 års alderen. Skrumplever kan videre utvikle seg til leverkreft. Man vet aldri hvem som er uheldig, det er derfor viktig å få kartlagt status på sykdommen.

Hvordan smitter hepatitt C

Smitten skjer i stor grad ved deling av brukerutstyr som f.eks. sprøyter, kokekar, filtre og hvor «blodsøl» er involvert. Viruset er mer overlevelsesdyktig enn HIV og kan overleve i flere dager på utstyr som har blitt liggende.
Å koke brukerutstyr gir ikke nok sikkerhet med tanke på smitte.
Bruk derfor alltid rent brukerutstyr!
Seksuell smitte er svært uvanlig. Men ved bruk av kondom kan du beskytte deg mot for eksempel HIV.
Barberhøvel og barbermaskin som er brukt av en med hepatitt C må ikke deles av andre i familien siden det kan innebære en risiko for blodsmitte.

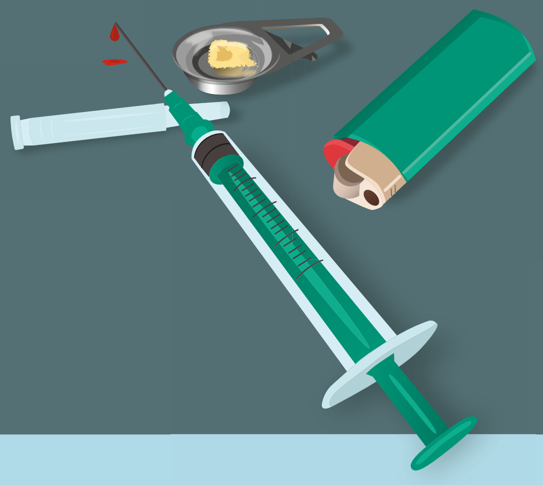

Å leve med hepatitt C

Dersom du har hepatitt C kan sunt kosthold og mosjon bidra til å holde leveren så frisk som mulig. Alkohol er svært skadelig og bør helst unngås dersom du har fått påvist alvorlig leverskade. Mange lever helt vanlige liv selv om de har hepatitt C. Det viktigste er at du er klar over det, og ikke smitter andre.

brosjyreHepCBergen.indd 2 07.04.2017 11:58:02
